# Noninvasive Methods to Evaluate Liver Fibrosis in Chronic HCV Infection

**DOI:** 10.5812/kowsar.1735143X.711

**Published:** 2011-09-01

**Authors:** Mortada Hassan El-Shabrawi, Mona Isa

**Affiliations:** 1 Department of Pediatrics, Faculty of Medicine, Cairo University, Cairo, Egypt

**Keywords:** Chronic hepatitis C, Liver fibrosis

Dear Editor,

Sirli et al.'s [1] effort to compare several noninvasive methods of fibrosis assessment in chronic hepatitis C virus (HCV) infection is commendable. Their study evaluated several simple serological tests for the prediction of fibrosis in chronic HCV infection: number of platelets, the aspartate aminotransferase-platelet ratio index (APRI test), the Forns score, the Lok score, and the FIB-4 score. They also compared these tests to liver stiffness measurement (LSM) by transient elastography (TE) and to the current "gold standard": liver biopsy (LB). They concluded that LSM was the best method for predicting cirrhosis, but all the evaluated tests had excellent predictive value [1]. We have to keep in mind that LSM failure can occur in 2%-10% of patients, and this is generally related to obesity, especially with the use of the M probe.

For almost all causes of chronic liver disease, assessment of fibrosis is important in estimating the prognosis of and determining the surveillance strategy for liver cancer. In addition, for chronic viral hepatitis, the degree of liver fibrosis is one important parameter for decisions of antiviral therapy. LB is still the standard and most commonly used procedure in the assessment of liver fibrosis. However, it is an invasive method associated with patient discomfort and in rare cases with serious complications. LBs are generally not performed in HCV patients with bleeding disorders because of increased bleeding risk and high costs. Antiviral treatment is only effective in 50% of these patients and is often accompanied by serious side effects. Consequently, careful selection of patients for treatment is warranted, and. assessment of liver fibrosis and cirrhosis noninvasively using LSM is an adequate alternative to LB [2].

The importance of using a combination of noninvasive tests for proper assessment of fibrosis in HCV patients has always been stressed. Recently, a series of algorithms based on sequential combination of noninvasive serum markers showed 93%-95% accuracy in the detection or exclusion of significant liver fibrosis and a reduction of 50% of liver biopsies in this subset of patients with HCV infection [3]. Pár A and Pár G concluded that both APRI and LSM results correlated with the METAVIR score, but LSM identified fibrosis better than APRI [4]. Likewise, Corradi and colleagues showed that LSM provides good accuracy in identifying patients with significant fibrosis and performs better than noninvasive indexes [5]. Similarly, Sirli et al. found that LSM by means of TE seems to be more sensitive than other noninvasive tests, including APRI for the prediction of significant fibrosis [1]. The advantage of APRI is that it is an easy, low-cost, and practical alternative for assessing structural changes in chronic HCV infection. Unfortunately, its performance is not reliable in females, younger patients, and in those with nonvertically transmitted HCV infection [6]. Alternatively, our group recommended the use of fibrotest (FT) as a noninvasive serum biomarker to assess the degree of hepatic fibrosis in pediatric patients with chronic HCV infection. At a cutoff value of 0.25, FT was able to discriminate patients with early-stage fibrosis from those with no or minimal fibrosis (sensitivity 92.3%, specificity 95.8%, and accuracy 94%). A higher cutoff (FT = 0.54) can be used to diagnose significant fibrosis (i.e., moderate or severe stages) with 71.4% sensitivity, 90.7% specificity, and 88.0% accuracy [7].

A significant drawback of noninvasive assessments of liver fibrosis is the inter- and intraobserver discrepancies in histological classification of lesser stages of fibrosis, which are more prevalent than for higher stages, and this may account for the observed underperformance of noninvasive tests when compared to histology as a reference standard [8]. In a study on more than 10,000 virtual biopsies, Bedossa et al. showed that liver fibrosis stage is correctly diagnosed in only 65% of cases if the biopsy is at least 15 mm long and in 75% of cases if it is at least 25 mm long. Additionally, Bedossa et al. found that the optimal size was 40 mm [9]. An important aspect of Şirli et al.'s study was that the researchers ensured that all of their LB fragments were at least 2 cm and included 8 portal tracts. This in turn ensured adequate pathological interpretation of the fibrosis stage and proper comparison with noninvasive tests.
